# Technology-Enabled Person-Centered Mental Health Services Reform: Strategy for Implementation Science

**DOI:** 10.2196/14719

**Published:** 2019-09-19

**Authors:** Haley M LaMonica, Tracey A Davenport, Katharine Braunstein, Antonia Ottavio, Sarah Piper, Craig Martin, Ian B Hickie, Shane Cross

**Affiliations:** 1 Brain and Mind Centre The University of Sydney Camperdown Australia; 2 InnoWell Pty Ltd Camperdown Australia

**Keywords:** implementation science, mental health, health care reform, technology, community-based participatory research

## Abstract

**Background:**

Health information technologies are being rapidly developed to improve the delivery of mental health care; however, a range of facilitators, barriers, and contextual conditions can impact the adoption and sustainment of these solutions. An implementation science protocol supports researchers to achieve primary effectiveness goals in relation to mental health services reform and aids in the optimization of implementation processes to promote quality health care, prolonging sustainability.

**Objective:**

The aim of this paper is to describe our implementation science protocol, which serves as a foundation by which to systematically guide the implementation of technology-enabled solutions in traditional face-to-face and Web-based mental health services, allowing for revisions over time on the basis of retrospective review and constructive feedback from the services in which the technology-enabled solutions are implemented.

**Methods:**

Our implementation science protocol comprises four phases. The primary objective of the scoping and feasibility phase (Phase 1) is to determine the alignment between the service partner and the quality improvement goals supported by the technology-enabled solution. This is followed by Phase 2, the local co-design and preimplementation phase, which aims to utilize co-design methodologies, including service pathway modelling, participatory design, and user (acceptance) testing, to determine how the solutions could be used to enhance the service. In Phase 3, implementation, the accepted solution is embedded in the mental health service to achieve better outcomes for consumers and their families as well as health professionals and service managers. Using iterative evaluative processes throughout Phase 3, the solution is continuously developed, designed, and refined during implementation to adapt to the changing needs of the stakeholders, including consumers with lived experience and their families as well as the service. Thus, the primary outcome of Phase 3 is the optimized technology-enabled solution that can be maintained in a service during the sustainment and scalability phase (Phase 4) for the purposes of mental health services reform.

**Results:**

Funding for the protocol was provided by the Australian Government Department of Health in June of 2017 for a period of 3 years. At the time of this publication, the protocol had been initiated in 11 services, serving three populations, all of which are currently operating in Phase 3. The first results are expected to be submitted for publication in 2020.

**Conclusions:**

With the aim of improving mental health service quality, our implementation science protocol aids in the identification of factors that predict the likelihood of implementation success, as well as the development of strategies to proactively mitigate potential barriers to achieve better implementation outcomes. Putting in place a theoretically sound implementation science protocol is essential to facilitate the uptake of novel technology-enabled solutions and evidence-based practices into routine clinical practice for the purposes of improved outcomes.

## Introduction

### Health Information Technologies and Mental Health Services Reform

Globally, the mental health system is plagued by fundamental shortcomings, including delays in service provision, limitations in access, fragmented services, failure to utilize routine outcome monitoring, and provision of care that does not match the consumer’s level of need. New health information technologies (HITs) are being rapidly developed to improve the delivery of mental health care for both health professionals and consumers, as well as to better support self-management of care. For example, cognitive-behavioral therapy approaches have been incorporated into several apps and websites, such as MoodMission [1] and CBT-i Coach [2], to help consumers better self-manage their health and well-being, provide psychoeducation about areas of concern or difficulty, and enhance traditional face-to-face care. Unfortunately, it is all too common for both traditional clinical and more novel electronic or Web-based interventions found to be efficacious in research studies not to be associated with meaningful outcomes for consumers in clinical settings [3]. This may partly relate to fundamental differences in the factors frequently associated with successful clinical trials (eg, highly standardized, homogenous participant sample, and control of possible confounding factors), relative to those that facilitate effectiveness in clinical practice or community settings (eg, flexibility in the intervention for providers and consumers, appropriateness for a broad consumer group across multiple clinical settings, and applicability for multiple conditions). However, it is also important to consider possible failures in the implementation of evidence-based interventions in practice. For example, a recent systematic review highlighted the failings of HIT to support the management of heart failure because of the complexities of providing care to consumers who are often older, with multiple comorbidities, more vulnerable with less support, and less technically savvy [4]. Furthermore, Bont and Bal describe an HIT as being set up for success by the clinical sponsors but failing because of the impact on traditional notions of what it meant to be a good health professional [5].

### Implementation of Health Information Technologies: Barriers and Facilitators

As it relates to health care, implementation science is defined as the scientific study of methods to promote the systematic uptake of research and development (R&D) findings and evidence-based practices into routine clinical practice, with the aim of improving the quality and effectiveness of health services and the care provided [6]. There is increasing appreciation among leading research organizations regarding the need to develop and utilize effective methods of implementing efficacious or effective interventions for the purposes of improving health care quality and efficiency [7-9]. To facilitate successful implementation, our implementation science protocol serves as a strategic, high-level, long-range plan with 3 primary aims: (1) describe the implementation process, (2) explain the facilitators and barriers to implementation outcomes, and (3) evaluate implementation [10].

#### Potential Facilitators and Barriers to Successful Implementation

The results of a recent systematic review highlighted the benefits of HITs on the quality and efficacy of health care, partly by facilitating adherence to guidelines or protocol-based care with the aid of embedded electronic decision support functions [11]. Specific examples from the mental health field include consistent support for phone calls or short message service text messages about medications, resulting both in improved adherence and reductions in hospitalizations, as well as for the use of Web-based self-management tools to improve quality of life, mood, and social functioning [12]. Handheld devices to support health care consultations have also been shown to improve satisfaction with care [12]. Despite the unique and additive benefits of HITs to mental health service quality, previous studies have shown that a range of facilitators, barriers, and contextual conditions impact on implementation adoption and sustainment [13-16], potentially resulting in underutilization. Specifically, several recent reviews have documented potential barriers and facilitators to implementation processes [17-21], which can be divided into service-level, health professional, and individual factors. Notably, there is often a reciprocal relationship between facilitators and barriers, such that a facilitator that is not championed can quickly become a barrier and vice versa.

#### Service Factors

The importance of leadership from the senior service management, as well as at the local service level, is consistently highlighted as a potential facilitator to successful implementation [17,18,21,22]. Indeed, our experience implementing a prototype technology-enabled solution [23], described in detail below, across 5 youth mental health services highlighted the benefit of strong local leadership to promote service innovation, as well as R&D within a service [24]. Senior leaders help ensure alignment between the technology and the service mission and help foster an organizational culture that is open to change in the service model and receptive to the technology-enabled solution [17]. In this regard, involvement of local champions (ie, prominent and well-respected individuals in a service) in the co-design and co-development of the solution and its implementation is frequently emphasized as a facilitator of successful implementation [18,25]. In addition, executive sponsorship is essential to emphasize the organizational commitment and demonstrate support for service change and redevelopment, especially given that the underestimation of change management can also act as a barrier to successful implementation [17,20]. Service leaders engender a positive attitude toward change and encourage adoption of the technology-enabled solution by frontline staff and consumers. Contingencies need to be put in place to ensure that the local champion roles are always filled, regardless of staff turnover.

Consideration should also be given to the degree of alignment between conventional service models and workflows and the solution, as misalignment represents a barrier to successful implementation, with higher degrees of misalignment increasing the complexity of the implementation [26]. Furthermore, in a systematic review of electronic health implementations, workflow disruptions were found to be the most cited factor in determining the success or failure of an implementation [20]. Collaboration and R&D between researchers and service staff (eg, health professionals, administrators, and service managers), as well as consumers with lived experience and their families, in relation to the iterative co-design and co-development of the technology-enabled solution (including service model), are critically important components to implementation success and sustainability for mental health services reform [15,18,20,27,28].

Resource limitations, including lack of appropriate personnel and equipment, are routinely reported as barriers to implementation [17,21]. Interestingly, however, the availability of adequate resources within a service has not been found to be a specific facilitator of implementation. As summarized by Vis et al [21], reliable access to information and communications technology (ICT), as well as any required interoperability with other existing technology within the service, such as an electronic medical record or client management system, is a key determinant of implementation.

#### Health Professional Factors

A number of factors related to health professionals have been identified as facilitators for successful implementation. For example, the co-design and configuration of the solution to fit the needs of health professionals foster buy-in and acceptance [18,21,22]. Successful implementation is also facilitated by effective education and training of health professionals, which nurtures self-efficacy and capacity in the context of continuous on-the-ground support [17,18,21,22]. Indeed, research indicates that learning outcomes are enhanced when initial education and training are supplemented with ongoing implementation support [29]. Several potential barriers to implementation at the level of health professionals have also been consistently reported in the literature, including negative staff attitudes, resistance to change, and changes to work practices [17,20-22].

#### Individual Factors

Implementation theories consistently state the importance of considering consumers’ needs when designing any implementation processes intended to improve outcomes [30,31]. As such, the involvement of consumers with lived experience and their families in the co-design process is key to implementation success [20]. Consideration of consumer preferences for and disparities in the use of digital devices and modes of technology (eg, email, app, and website) to connect with health services is essential [32] when iteratively designing and developing technology-enabled solutions. Other identified facilitators include consideration of the convenience and appropriateness of the solution in addressing consumers’ health care needs [21], the user friendliness of the solution [22,33], and the appropriate adaptations (ie, configuration) of the solution to fit the specific cultural needs of the populations seeking care [18]. As drivers of acceptance [21,22] and empowerment for consumers with lived experience [20], the combination of these factors is likely to promote successful implementation. Conversely, individual resistance or nonparticipation, recruitment and retention issues, individual concerns about privacy, confidentiality, and information security, and a failure to adapt solutions over time to meet individual needs [17,20,22] are consistent barriers to successful implementation and, in turn, mental health services reform.

### Co-Design of the Technology-Enabled Solution

As described in detail in a study by Davenport et al [23], the co-designed InnoWell Platform was developed through Project Synergy (by InnoWell Pty Ltd) to collect, store, and report clinical data back to a consumer and their health professional to promote person-centered care, self-management, early intervention, shared decision making, and routine outcome monitoring (see Textbox 1).

**Description of the InnoWell Platform as it is listed on the Australian Register of Therapeutic Goods (software as a medical device, class 1, ARTG ID 315030).**

The InnoWell Platform is a customizable digital tool that assists assessment, monitoring and management of mental health issues, and maintenance of wellbeing. It does this by collecting personal and health information from consumers and their service providers. This information is stored, scored, and reported back to consumers and their health professionals to promote collaborative care. The clinical content is determined in collaboration with the service provider who invited the consumer to use the platform. Importantly, the platform does not provide stand-alone medical or health advice, diagnosis, or treatment. Instead, it guides and supports (but does not direct) consumers and their health professionals to decide what may be suitable care options. Importantly, all care aligns with the existing clinical governance (eg, policies and procedures) of the service provider.

## Methods

### An Implementation Science Protocol for Local Mental Health Services Reform

For the purposes of the technology-enabled solution, quality implementation is achieved when the deployment of the technology and service model into health care services meets the requirements and standards to achieve the desired outcome [34], namely technology-enabled mental health services reform. With this aim in mind, our implementation science protocol incorporates elements from 3 sources, namely the Quality Implementation Framework [29] and the Accelerated Creation-to-Sustainment model [15], as well as learnings from our experience co-designing and implementing a prototype technology-enabled solution into primary mental health care settings with young people [24,35]. The primary objective is to design a standardized yet flexible implementation science protocol to serve as a foundation by which to systematically guide implementation efforts, allowing for revisions over time on the basis of retrospective review and constructive feedback from services in which the technology-enabled solution is to be implemented and the consumers who will be engaging with the solution. Our implementation science protocol comprises 4 phases. The objectives and outcomes of each phase are described in detail below.

#### Phase 1: Scoping and Feasibility

The primary objective of Phase 1 is to determine the fit between the aims of the potential mental health service partner and the quality improvement goals supported by the technology-enabled solution. To begin with, it is essential to ensure that the service leadership is invested in quality improvement at the individual level and to demonstrate the relationship between this aim and the components of the technology-enabled solution. Our protocol emphasizes the importance of engaging organizational leaders from the earliest steps of the process. Furthermore, it is crucial to understand the basic attributes of the service, including the following: the characteristics of the consumers who access the service for care, the qualifications and occupancy of the service health professionals, administrators and service managers, and the current hardware and ICT infrastructure (eg, electronic medical record, client management system, and availability of Wi-Fi). It is also important to identify those persons who will be responsible for facilitating the implementation process at the service, including making decisions related to configuration and customization of the technology-enabled solution and overseeing change management processes (eg, changes to the clinical pathway, user journey, and workflows) on the ground. Provided the key tasks outlined in the scoping and feasibility phase are addressed, transition to Phase 2 then occurs. Alternatively, the service may choose to address the internal service gaps identified in this phase before proceeding to the next phase.

#### Phase 2: Co-Design and Preimplementation

A key feature of the technology is that the functionality and content are configurable, which allows it to adapt easily to local contexts, as well as specialist clinical and population groups, and it thus meets the person-centered care needs of a wider range of people presenting to diverse mental health providers. The primary objective of this phase is to utilize novel and innovative co-design methodologies to develop the technology-enabled solution and service model, as well as to user (acceptance) test these solutions for mental health services reform.

##### Participatory Design of the Technology Solution

**Figure 1:**
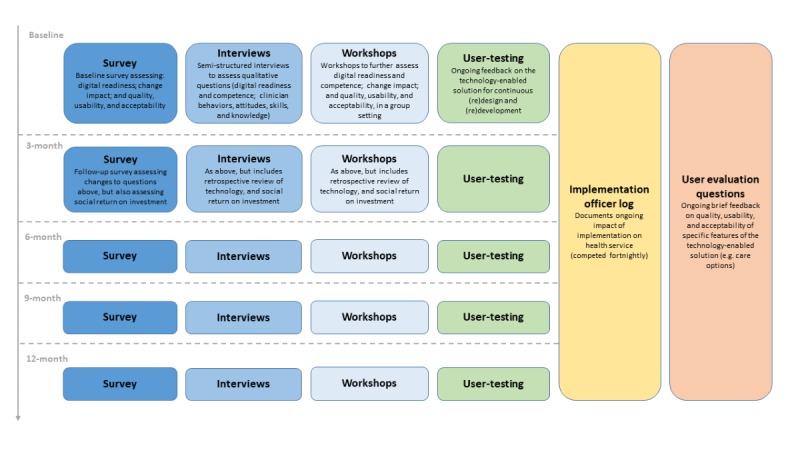
Flow diagram of evaluative methods and processes to optimize technology-enabled solutions for mental health services reform.

As previously described in detail by our team [35-37], the potential end users (eg, general population, consumers with lived experience and their families, health professionals, administrators, and service managers) help inform the development of the technology through the continuous and iterative use of participatory design (or co-design), knowledge translation, user (acceptance) testing, and rapid prototyping methodologies. These user-centered methodologies emphasize the involvement of individuals with lived experienced (eg, consumers, family members, and peer support workers) in the co-design process of the technology and the implementation process. As outlined by Mohr et al [15], interactive and iterative participatory design methodologies with key stakeholders (eg, information gathering, clarification of user requirements, workflow observations, co-design workshops, and user testing) help adapt the technology to local needs. Furthermore, as outlined in Figure 1, our protocol incorporates iterative evaluation methods to promote the continuous development and design of the technology, as well as the implementation process for the service and consumers for whom they provide care. The process of refining the technology can occur iteratively through the Implementation and Sustainment phases [15].

##### Co-Designing Technology-Enabled Service Models

**Figure 2:**
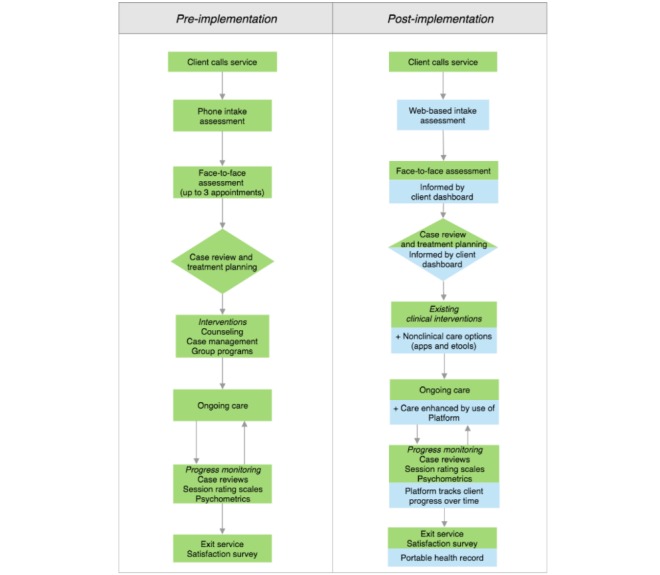
Hypothetical service models showing pre and post implementation of the technology-enabled solution.

In parallel, a process of co-designing service models occurs with representative stakeholders. The preexisting service model and related staff roles and responsibilities are mapped by using a process of service modelling developed through R&D. As illustrated in the hypothetical example shown in Figure 2, each aspect of an individual’s journey through the service is delineated, starting from the perspective of the individual seeking help and expanding when other service stakeholders interact with that individual through the care journey. Following the presentation of an individual’s experience of a prototypic version of the technology-enabled solution, the gaps between the current model offered by the service and the key features of high-quality mental health care are explored. A hypothesized technology-enhanced service model is then co-designed, intertwining established processes with the additional technology elements and processes (eg, Web-based intake assessment, video visit). The changes to workflows and practices for each of the affected stakeholders (eg, health professional, administrator, and service manager) are also noted throughout the model. Finally, existing service metrics used to report on service quality are reviewed (as available) across pre-established quality domains (eg, safety, efficiency, and accessibility); thereafter, agreement is reached on the metrics used to monitor service quality using the technology solution [38].

##### Education and Training

The education and training needs of service staff across all roles (eg, health professionals, administrators, and service managers), as well as consumers, are scoped during Phase 2. First, education and training are provided in relation to the evidence-based digital, clinical, service, and safety elements essential to improving service quality. Second, training in the use of the technology-enabled solution includes all necessary information to use the technology effectively for all staff members, including both a comprehensive overview of the functionality and the components relevant to the roles in the service. As outlined in Table 1, education and training are provided before implementation, with ongoing education, training, and technical assistance available throughout Phase 3.

##### Discovering Facilitators and Barriers Before Implementation

In Phase 2, ongoing interactions with the team of stakeholders involved in the implementation and change management process, present opportunities for the active and passive identification of barriers to, and facilitators of, successful implementation not previously uncovered. Table 1 highlights some of the potential barriers identified in previous studies and the mitigation strategies for each.

**Table 1:** Potential service-specific barriers and mitigation strategies to implementation of our technology-enabled solutions.

Barriers	Mitigation strategies
Negative staff attitudes [20,21]; staff resistance to change [17,20-22]; changes to work practices, such as increased workload [20-22]	Engagement and buy-in fostered through participatory design and user (acceptance) testing; co-design of service model to identify gaps between new and existing workflows and foster collaborative problem-solving approaches to customization and configuration of the technology-enabled solution for the benefit of service quality improvement; development of a communication strategy to assist the service with messaging within internal stakeholders and service users
Staff turnover and lack of staff resources [17,18]	Implementation Officer embedded in service to maintain continuity of support; contingency plan for training new staff
Innovation not able to adapt over time to meet staff needs [17,20,22]	Ongoing evaluation of the technology-enabled solution; continuous and iterative refinement of the technology-enabled solution and service model throughout Phases 3 and 4 (implementation and sustainment)
Design and usability of the technology-enabled solution [20,21,24]; adaptability/flexibility of the technology-enabled solution [17,18,22]; compatibility/fit of technology-enabled solution with service mission [17,18]; user resistance to the technology-enabled solution [22]; noninteroperability (or limited) with other information and communications technology systems [17,20]; fidelity of implementation [17,22]; availability of and user familiarity with required equipment to use the technology [21,24]	Service provider readiness assessment to determine compatibility of the technology-enabled solution with the service; co-design for a service-specific technology-enabled solution; technology configuration and customization arising from the co-design process, including co-design and co-development of service-specific content, as well as integration with service information and communications technology systems; iterative user experience and user acceptance testing; iterative evaluative processes related to technology and implementation process, highlighting adaptability of the technology; provision of ongoing education and training and technical assistance

#### Phase 3: Implementation

Phases 1 and 2 are seen as critical to successful implementation, as they foster buy-in and commitment from stakeholders to the principles of co-design and quality improvement, and they should lead to a point where all stakeholders feel heard and are able to participate in the change process. As far as possible, all known barriers and facilitators to implementation, as they apply to the local context, have been identified at this stage, with a mitigation strategy identified for each. Despite this, new barriers and facilitators may be uncovered, which were previously unknown or underestimated; thus, having clearly established processes and identified persons to support the service through the change is key to address issues as they arise. Providing on-the-ground support to service users and staff is valuable in the early weeks or months of implementation. Furthermore, gathering feedback from all user groups, as they use the technology-enabled solution in practice, refines the technology and service model, but more importantly, the workforce and structural changes required to improve service quality (Figure 2). As illustrated in Figure 1, ongoing feedback from service staff (eg, health professionals, administrators, and service managers) is collected via Web-based surveys, semistructured interviews, and workshops to evaluate and monitor the impact of embedding the technology-enabled solution in the service, including (1) service-level changes in outcomes and processes, as well (2) as the digital readiness and competence of service staff, (3) quality, usability, and acceptability of the solution, and (4) social return on investment of embedding the solution in the service. In addition, feedback from both staff and consumers about existing and newly designed functionality is captured through quarterly user testing sessions. The fortnightly Implementation Officer Logs are also used to gather data from service staff, including anonymous commentary and feedback provided by individuals with lived experience, including consumers and their families or supportive others, who are engaging with the solution as part of care, which are then fed back through to R&D processes to inform the iterative design and development of the technology-enabled solution. This feedback may include technical difficulties, as well as comments in relation to the user experience and clinical aspects of the solution. Google Analytics is also embedded within the solution to allow back-end analysis of user behavior, including details regarding the features of solution that consumers use most consistently, as well as those features with which they disengage most quickly. Of note, in the absence of a 24-hour monitoring protocol to ensure the safety of all users at this time, there is no option for consumers to provide free text feedback.

Importantly, it is generally accepted in digital mental health research that the technology-enabled solution will be iteratively developed, designed, and refined during implementation. The expected outcome of Phase 3 is for the technology to be embedded and integrated within the service, such that it is seen as a vital piece of standard care, enabling and maintaining ongoing service quality improvement and reform.

#### Phase 4: Sustainment and Scalability

In accordance with Lennox et al [39], the primary objective of Phase 4 is the continuation and maintenance of our technology-enabled solutions and their associated outcomes within a health service, as well as the iterative process of evaluation and design, to address problems and emerging needs and demands of the service, individual populations, and the broader context [15]. To achieve this aim, prospective approaches are employed throughout the preceding phases to build relationships with and foster buy-in from key stakeholders (ie, consumers with lived experience and their families, health professionals, administrators, and service managers), as well as to iteratively design and refine the technology-enabled solution to adapt to the changing needs of the stakeholders and service [39]. Employing these processes helps to ensure the continued effectiveness of the technology-enabled solution, including improved access to care and resources to promote mental health and well-being, the integration of the technology within the service, and community ownership of the solution [40]. When taken together, this reflects the primary outcome of Phase 4, namely technology-enabled mental health services reform.

Furthermore, the objective of Phase 4 is to leave a configurable technology-enabled solution in place for ongoing and continued benefit to the service, following the replacement of a locally available Implementation Officer with a more sustainable supporting resource that is readily available remotely.

### Participating Centers

At the time of this publication, the implementation science protocol had gone live in the following participating centers: headspace services (Ashfield, Camperdown, Coffs Harbour, Hurstville, Lismore, Port Macquarie, Miranda, and Tweeds Head, New South Wales, and Edinburgh North, South Australia), Butterfly’s National Helpline, and Open Arms—Veterans and Families Counselling Surry Hills, New South Wales.

### Sample Size

The protocol phases do not have an upper or lower limit on the number of participants, as this will vary by participating center, both in relation to the number of staff members (eg, health professionals, administrators, and service managers) and the size and diversity of their consumer base.

### Data Analyses

Qualitative and quantitative data analyses will be conducted to assess the success of the implementation at the level of the consumer, health professional, and service, and, where possible, comparative analyses will be run between and within participating centers and populations, to allow for the identification of commonalities and differences in outcomes.

### Ethics

Ethics approvals to conduct all aspects of the protocol are sought from the relevant governing Human Research Ethics Committees for the participating centers.

## Results

At the time of this publication, all participating centers were in Phase 3. The first results from Phases 1 to 3 are expected to be submitted for publication in 2020, with Phase 4 data expected thereafter.

## Discussion

The international goal of substantially improving the quality of mental health services is central to many technology-based innovation implementation efforts in mental health service delivery. As seen in a range of other industries, technology-enabled disruption brings with it significant changes to conventional practice and experience for all stakeholders. The greater the gap between the innovation and conventional practice, the greater the implementation challenge and, arguably, the greater the need for technology and service co-design with all stakeholders. This protocol incorporates the findings that affect implementation success in the rapidly evolving implementation science literature, to serve as both preemptive mitigation strategies and foci for surveillance throughout each of the implementation phases. With the aim of avoiding obsolescence of the solutions, our implementation science protocol also stresses the parallel and iterative evaluation of the effectiveness of the technology-enabled solution alongside the success, or lack thereof, of the implementation.

## Abbreviations

BMC: Brain and Mind Centre
HIT: health information technology
ICT: information and communications technology
R&D: research and development

## References

[1] (2016). iTunes - Apple.

[2] (2013). iTunes - Apple.

[3] Glasgow RE, Lichtenstein E, Marcus AC (2003). Why don't we see more translation of health promotion research to practice? Rethinking the efficacy-to-effectiveness transition. Am J Public Health.

[4] Greenhalgh T, A'Court C, Shaw S (2017). Understanding heart failure; explaining telehealth - a hermeneutic systematic review. BMC Cardiovasc Disord.

[5] de Bont A, Bal R (2008). Telemedicine in interdisciplinary work practices: on an IT system that met the criteria for success set out by its sponsors, yet failed to become part of every-day clinical routines. BMC Med Inform Decis Mak.

[6] Eccles MP, Mittman BS (2006). Welcome to implementation science. Implement Sci.

[7] (2012). Centers for Disease Control and Prevention.

[8] (2000). National Health and Medical Research Council.

[9] (2018). The National Institute of Mental Health.

[10] Nilsen P (2015). Making sense of implementation theories, models and frameworks. Implement Sci.

[11] Chaudhry B, Wang J, Wu S, Maglione M, Mojica W, Roth E, Morton SC, Shekelle PG (2006). Systematic review: impact of health information technology on quality, efficiency, and costs of medical care. Ann Intern Med.

[12] Lawes-Wickwar S, McBain H, Mulligan K (2018). Application and effectiveness of telehealth to support severe mental illness management: systematic review. JMIR Ment Health.

[13] Feijt MA, de Kort YA, Bongers IM, IJsselsteijn WA (2018). Perceived drivers and barriers to the adoption of emental health by psychologists: the construction of the levels of adoption of emental health model. J Med Internet Res.

[14] Ross J, Stevenson F, Lau R, Murray E (2015). Exploring the challenges of implementing e-health: a protocol for an update of a systematic review of reviews. BMJ Open.

[15] Mohr DC, Lyon AR, Lattie EG, Reddy M, Schueller SM (2017). Accelerating digital mental health research from early design and creation to successful implementation and sustainment. J Med Internet Res.

[16] Hermes ED, Lyon AR, Schueller SM, Glass JE (2019). Measuring the implementation of behavioral intervention technologies: recharacterization of established outcomes. J Med Internet Res.

[17] Bach-Mortensen AM, Lange BC, Montgomery P (2018). Barriers and facilitators to implementing evidence-based interventions among third sector organisations: a systematic review. Implement Sci.

[18] Durlak JA, DuPre EP (2008). Implementation matters: a review of research on the influence of implementation on program outcomes and the factors affecting implementation. Am J Community Psychol.

[19] Geerligs L, Rankin NM, Shepherd HL, Butow P (2018). Hospital-based interventions: a systematic review of staff-reported barriers and facilitators to implementation processes. Implement Sci.

[20] Granja C, Janssen W, Johansen MA (2018). Factors determining the success and failure of ehealth interventions: systematic review of the literature. J Med Internet Res.

[21] Vis C, Mol M, Kleiboer A, Bührmann L, Finch T, Smit J, Riper H (2018). Improving implementation of emental health for mood disorders in routine practice: systematic review of barriers and facilitating factors. JMIR Ment Health.

[22] Greenhalgh T, Wherton J, Papoutsi C, Lynch J, Hughes G, A'Court C, Hinder S, Fahy N, Procter R, Shaw S (2017). Beyond adoption: a new framework for theorizing and evaluating nonadoption, abandonment, and challenges to the scale-up, spread, and sustainability of health and care technologies. J Med Internet Res.

[23] Davenport TA, LaMonica HM, Whittle L, English A, Iorfino F, Cross S, Hickie IB (2019). Validation of the InnoWell platform: protocol for a clinical trial. JMIR Res Protoc.

[24] Cross S, Piper S, Davenport TA, Milton A, Iorfino F, Ricci C, Ospina-Pinillos L, Whittle L, Hickie IB (2019). Implementation trial of a prototypic e-clinic within youth mental health services: staff experiences and reported service quality improvements. Med J Aust.

[25] Boswell JF, Kraus DR, Miller SD, Lambert MJ (2015). Implementing routine outcome monitoring in clinical practice: benefits, challenges, and solutions. Psychother Res.

[26] Nazi KM (2013). The personal health record paradox: health care professionals' perspectives and the information ecology of personal health record systems in organizational and clinical settings. J Med Internet Res.

[27] Ceptureanu S, Ceptureanu E, Luchian C, Luchian I (2018). Community based programs sustainability. A multidimensional analysis of sustainability factors. Sustainability.

[28] Ben-Tovim DI, Dougherty ML, O'Connell TJ, McGrath KM (2008). Patient journeys: the process of clinical redesign. Med J Aust.

[29] Meyers DC, Durlak JA, Wandersman A (2012). The quality implementation framework: a synthesis of critical steps in the implementation process. Am J Community Psychol.

[30] Damschroder LJ, Aron DC, Keith RE, Kirsh SR, Alexander JA, Lowery JC (2009). Fostering implementation of health services research findings into practice: a consolidated framework for advancing implementation science. Implement Sci.

[31] Feldstein AC, Glasgow RE (2008). A practical, robust implementation and sustainability model (PRISM) for integrating research findings into practice. Jt Comm J Qual Patient Saf.

[32] Gordon NP, Hornbrook MC (2016). Differences in access to and preferences for using patient portals and other ehealth technologies based on race, ethnicity, and age: a database and survey study of seniors in a large health plan. J Med Internet Res.

[33] van Dyk L (2014). A review of telehealth service implementation frameworks. Int J Environ Res Public Health.

[34] Meyers DC, Katz J, Chien V, Wandersman A, Scaccia JP, Wright A (2012). Practical implementation science: developing and piloting the quality implementation tool. Am J Community Psychol.

[35] Davenport TA, Milton A, Ospina-Pinillos L, Whittle L, Ricci CS, Burns JM, Hickie IB (2019). The research and development cycle for project Synergy: an iterative process of participatory design, user testing and real-world trialing. Med J Aust.

[36] Ospina-Pinillos L, Davenport TA, Ricci CS, Milton AC, Scott EM, Hickie IB (2018). Developing a mental health eclinic to improve access to and quality of mental health care for young people: using participatory design as research methodologies. J Med Internet Res.

[37] LaMonica HM, Davenport T, Burns J, Cross S, Hodson S, Veitch J, Hickie IB (2019). Mental health service reform for open arms–veterans and families counselling: a participatory design study. JMIR Form Res.

[38] Cross S, Davenport TA, LaMonica HM, Hickie IB (2019). Potential of real-time and integrated clinical data to drive continuous quality improvement in youth mental health services. Med J Aust.

[39] Lennox L, Maher L, Reed J (2018). Navigating the sustainability landscape: a systematic review of sustainability approaches in healthcare. Implement Sci.

[40] (2004). World Health Organization.

